# Beyond the MNA: A Biological Vulnerability Phenotype Associated with Prolonged Hospitalization in Older Adults

**DOI:** 10.3390/nu18040586

**Published:** 2026-02-11

**Authors:** Virginia Boccardi, Francesca Mancinetti, Cesare Balducci, Valeria Labbozzetta, Elena Ercolanetti, Carmelinda Ruggiero, Sara Ercolani, Patrizia Mecocci

**Affiliations:** 1 Division of Gerontology and Geriatrics, Department of Medicine and Surgery, University of Perugia, 06123 Perugia, Italy; francesca.mancinetti@dottorandi.unipg.it (F.M.); elena.ercolanetti@ospedale.perugia.it (E.E.); sara.ercolani@ospedale.perugia.it (S.E.); patrizia.mecocci@unipg.it (P.M.); 2 Division of Clinical Geriatrics, Department of Neurobiology, Care Sciences and Society, Karolinska Institute, 17177 Stockholm, Sweden

**Keywords:** hospitalization, hypoalbuminemia, inflammation, malnutrition screening, older adults

## Abstract

Background/Objectives: Nutritional status in hospitalized older adults is usually evaluated using screening tools such as the Mini Nutritional Assessment (MNA). During acute illness, however, these tools may overlook biologically relevant vulnerability related to inflammation and metabolic stress, a condition often described as occult malnutrition. The clinical implications of applying a more stringent biological definition of this phenotype are still poorly defined. Methods: We conducted an observational cohort study including 172 hospitalized older adults (mean age 86.7 ± 6.0 years) in an acute geriatric setting with complete data on MNA, serum albumin, hemoglobin, and C-reactive protein (CRP). Occult malnutrition was defined by the coexistence of at least two abnormal biological markers: hypoalbuminemia (albumin < 3.5 g/dL), anemia (Hb < 12 g/dL), and elevated CRP (>0.5 mg/dL). Prolonged length of stay (LOS) was defined as LOS above the 75th percentile. Associations between occult malnutrition, MNA categories, and prolonged LOS were examined using chi-square tests and multivariable logistic regression models adjusted for age ≥ 85 years, sex, primary reason for hospital admission and comorbidity burden. Results: Among 172 acutely hospitalized older adults (mean age 86.7 ± 6.0 years), hypoalbuminemia, anemia, and elevated CRP were observed in 42.4%, 46.5%, and 72.1% of patients, respectively. Occult malnutrition was identified in 54.7% of the cohort. Prolonged length of stay (LOS > 75th percentile) occurred in 50.0% of patients. Compared with patients without occult malnutrition, those with occult malnutrition were older, had significantly longer hospital stays, and a higher prevalence of prolonged LOS (59.6% vs. 38.5%, *p* = 0.006). The prevalence of occult malnutrition increased across worsening MNA categories (*p* = 0.022) and was present in 40.7% of patients with a normal MNA score. Occult malnutrition was independently associated with prolonged LOS in multivariable models adjusting for age, sex, admission reason, comorbidity burden, and MNA (adjusted OR range 1.97–2.14, all *p* < 0.05), whereas MNA itself was not independently associated with prolonged hospitalization. Conclusions: A stringent biological definition of occult malnutrition identifies a large subgroup of hospitalized older adults at increased risk of prolonged hospitalization, including patients considered nutritionally normal by standard screening. These findings underline the limitations of screening-based assessment alone and support the use of simple biological markers to refine risk stratification in acute geriatric care.

## 1. Introduction

Malnutrition is highly prevalent yet frequently under-recognized among hospitalized older adults and is consistently associated with adverse clinical outcomes, including prolonged hospitalization, functional decline, and increased mortality [1,2,3]. In acute care settings, nutritional screening tools such as the Mini Nutritional Assessment (MNA) are widely recommended to identify older patients at risk and to guide nutritional interventions [4,5]. However, these instruments are primarily related to anthropometric measures, dietary intake, and functional indicators and may fail to adequately capture the underlying biological vulnerability associated with acute illness and systemic inflammation.

In older adults, acute hospitalization is often characterized by a complex interplay between inflammation, catabolism, and metabolic dysregulation [6,7]. This biological response may manifest as laboratory abnormalities such as hypoalbuminemia, anemia, and elevated inflammatory markers, even in patients who do not meet conventional criteria for malnutrition according to screening tools. Increasing evidence suggests that these biological alterations reflect reduced physiological reserve and impaired stress response rather than nutritional deficiency alone [8,9,10].

The concept of occult malnutrition is here proposed as an exploratory framework to describe the discrepancy between apparently normal nutritional screening results and underlying biological vulnerability. Within this framework, malnutrition is not defined solely by weight loss or dietary intake but also by the biological response to illness, including inflammatory activation, catabolic drive, and impaired protein synthesis [5]. In this context, we use the term ‘occult malnutrition’ as a composite to describe a vulnerability phenotype characterized by inflammatory activation and reduced physiological reserve during acute illness, rather than as a formal nutritional diagnosis. Existing evidence primarily supports the prognostic value of individual biomarkers [11,12], and their combined use in a composite definition is proposed here as an operational approach to capture biological vulnerability during acute illness. This approach does not aim to replace established diagnostic criteria such as GLIM [5] but rather to complement conventional screening tools by capturing disease- and inflammation-related vulnerability that may remain clinically occult. This paradigm is particularly relevant in geriatric populations, in whom chronological age poorly reflects biological resilience and recovery potential [13]. To date, no validated composite definition integrating these routinely available biological markers has been proposed to capture this form of subclinical nutritional vulnerability in hospitalized older adults. Consequently, the clinical implications of occult malnutrition remain largely unexplored. It is unclear whether this biological vulnerability provides prognostic information beyond established nutritional screening tools or whether it is independently associated with clinically meaningful outcomes, such as prolonged length of stay.

Therefore, this study aimed to: (i) describe the prevalence of occult malnutrition among hospitalized older adults with available MNA data; and (ii) investigate the association between occult malnutrition and length of stay, both in the overall cohort and in the subgroup of patients classified as nutritionally normal according to MNA.

## 2. Materials and Methods

### 2.1. Study Design and Population

The study population represents a real-world cohort of hospitalized older adults admitted to an acute geriatric care setting during six months. As part of routine clinical practice, all patients underwent a comprehensive multidimensional geriatric assessment whenever clinically feasible, including nutritional screening using the Mini Nutritional Assessment (MNA). Among 203 hospitalized patients, complete MNA data were available for 172 individuals, who constituted the study population. Incomplete MNA data were primarily due to clinical instability, severe cognitive impairment, early discharge, or transfer before completion of the geriatric assessment. Patients admitted for active oncologic disease or receiving ongoing cancer-specific treatments were excluded. The research protocol adhered to the ethical standards outlined in the Declaration of Helsinki and followed the principles of Good Clinical Practice. Ethical approval was obtained from the Ethics Committee of the University of Perugia (Protocol No. CE-685/24).

### 2.2. Clinical Assessment

Clinical information was collected through medical history and a comprehensive geriatric multidimensional evaluation at hospital admission as previously described [14]. Nutritional status was assessed using the MNA. According to standard cut-offs, patients were categorized as malnourished (MNA < 17), at risk of malnutrition (MNA 17–23.5), or with a non-pathological nutritional status (MNA ≥ 24). Patients with MNA ≥ 24 were considered nutritionally normal based on conventional screening criteria. Functional status was evaluated at hospital discharge using standardized measures of basic and instrumental activities of daily living. Activities of Daily Living (ADL) and Instrumental Activities of Daily Living (IADL) scores were recorded and expressed as mean ± standard deviation. Cognitive performance was assessed using the Mini-Mental State Examination (MMSE), with scores corrected for age and education according to validated norms. Depressive symptoms were evaluated using the Geriatric Depression Scale (GDS). Comorbidity burden was assessed using the Cumulative Illness Rating Scale for Geriatrics (CIRS-G). Both the cumulative score and the severity index were calculated. Trained healthcare professionals performed all functional and cognitive assessments in accordance with routine geriatric evaluation protocols. The primary clinical outcome was prolonged length of stay, defined as hospitalization exceeding the 75th percentile.

### 2.3. Categorization of the Primary Reason for Hospital Admission

The primary reason for hospital admission was extracted from medical records as a free-text variable. To enable analytical use while preserving clinical interpretability, this variable was systematically categorized using a predefined keyword-based algorithm. Text entries were standardized (lowercase conversion and trimming), and non-informative values (empty strings or numeric placeholders) were treated as missing. Based on geriatric clinical relevance and anticipated impact on inflammatory burden and hospital course, admissions were grouped into six mutually exclusive categories: (1) Fall; (2) Infectious/Febrile/Sepsis-related; (3) Cardio-respiratory (non-infectious); (4) Neurological/Cognitive/Syncope/Vertigo-related; (5) Gastrointestinal/Bleeding/Abdominal symptoms; (6) Metabolic/Renal/Anemia/Failure-to-thrive or other causes. This categorized variable was included as an adjustment covariate in the main multivariable logistic regression model to account for heterogeneity in acute illness presentation and as a proxy for disease severity. This variable was intended to capture the clinical context of acute hospitalization rather than disease severity per se and should be interpreted as an adjustment for diagnostic heterogeneity rather than a severity score. When multiple admission-related terms were present, priority was assigned based on the primary clinical driver of hospitalization as documented in the medical record, following a predefined hierarchical rule. Representative classification examples and a schematic decision flowchart are provided in the Supplementary Materials to enhance reproducibility.

### 2.4. Biological Parameters

In accordance with standard clinical practice in the acute geriatric setting, serum albumin, hemoglobin, and C-reactive protein were typically measured at hospital admission or within the first 24–48 h of hospitalization as part of the initial clinical evaluation. Serum albumin levels (g/dL) were analyzed as a continuous variable, and hypoalbuminemia was defined as albumin < 3.5 g/dL. Anemia was defined as hemoglobin levels < 12 g/dL. Systemic inflammation was assessed using C-reactive protein (CRP), with elevated CRP defined as >0.5 mg/dL. A single hemoglobin cut-off (<12 g/dL) was used for both sexes to reflect biological vulnerability in the acute geriatric setting rather than sex-specific hematological diagnoses. This choice was driven by the exploratory nature of the study and the predominance of female patients, and its limitations are acknowledged. Albumin was used as a marker of biological vulnerability rather than nutritional status, as its levels are influenced by inflammation and acute physiological stress.

Occult malnutrition was operationalized for analytical purposes as a composite indicator of biological vulnerability and should be interpreted as an exploratory construct rather than a validated diagnostic category. The requirement for at least two abnormal biomarkers was chosen as an operational criterion to increase specificity for a consolidated state of biological vulnerability, rather than to attribute prognostic weight to any single parameter. This definition was not intended to distinguish nutritional deficiency from inflammation or disease severity but to capture their coexistence during acute illness.

### 2.5. Statistical Analysis

Continuous variables are presented as mean ± standard deviation, and categorical variables as number (percentage). The main analyses evaluating the association between occult malnutrition and clinical outcomes were conducted in the overall cohort. Exploratory analyses focusing on discordance between nutritional screening and biological vulnerability were restricted to patients with non-pathological MNA scores (MNA ≥ 24). Associations between occult malnutrition and categorical outcomes were explored using contingency tables, chi-square or Fisher’s exact tests, as appropriate, and expressed as odds ratios with 95% confidence intervals. Bivariate associations between serum albumin, hemoglobin, C-reactive protein, and length of hospital stay were explored using correlation analysis. Multivariable logistic regression was used to assess independent associations with prolonged length of stay. The final model was constructed to evaluate whether the association between occult malnutrition and prolonged length of stay was independent of global comorbidity burden. The cumulative CIRS-G score was included to account for multimorbidity and was standardized (z-score) to reduce collinearity and improve model stability. An interaction term between CIRS-G and admission reason was tested to assess potential effect modification. Given the exploratory nature of the study, no multiple-testing adjustment was performed. Statistical significance was set at a two-sided *p*-value < 0.05.

## 3. Results

### 3.1. Study Population and Biological Abnormalities

In this cohort (N = 172), hypoalbuminemia (albumin < 3.5 g/dL) was observed in 73/172 (42.4%), anemia (Hb < 12 g/dL) in 80/172 (46.5%), and elevated CRP (>0.5 mg/dL) in 124/172 (72.1%). Occult malnutrition was identified in 94/172 (54.7%), whereas 78/172 (45.3%) showed 0–1 abnormal marker. Prolonged length of stay (LOS > 75th percentile) occurred in 86/172 (50.0%) patients. Fall-related conditions were the most frequent cause of admission (32.7%), followed by metabolic, renal, anemia-related, or failure-to-thrive conditions (19.0%). Neurological and cognitive presentations accounted for 14.3% of admissions, while cardiorespiratory causes not related to infection accounted for 13.7%. Infectious or febrile conditions were observed in 11.3% of cases, and gastrointestinal or bleeding-related symptoms in 8.9%.

Compared with patients without occult malnutrition, those with occult malnutrition were older (87.8 ± 5.6 vs. 85.5 ± 6.2 years, *p* = 0.010) and experienced a significantly longer hospital stay (14.3 ± 10.3 vs. 10.3 ± 6.2 days, *p* = 0.002), with a higher prevalence of prolonged LOS (59.6% vs. 38.5%, *p* = 0.006). Patients with occult malnutrition also exhibited lower MNA score (20.6 ± 3.7 vs. 21.9 ± 3.4, *p* = 0.017) and a higher proportion of malnutrition or risk of malnutrition according to MNA categories (*p* = 0.013). As expected, they showed markedly worse biological profiles. From a functional perspective, patients with occult malnutrition had lower ADL scores at discharge (2.49 ± 2.13 vs. 3.27 ± 2.09, *p* = 0.018), whereas no significant differences were observed in IADL, cognitive performance (corrected MMSE), or depressive symptoms (GDS). Measures of comorbidity burden, including the cumulative CIRS-G score and the CIRS severity index, tended to be higher in patients with occult malnutrition, although these differences did not reach conventional levels of statistical significance (both *p* = 0.055). Overall, baseline demographic, clinical, functional, and biological characteristics of the study population stratified by occult malnutrition status are summarized in Table 1.

### 3.2. Occult Malnutrition Across MNA Categories

**Figure 1:**
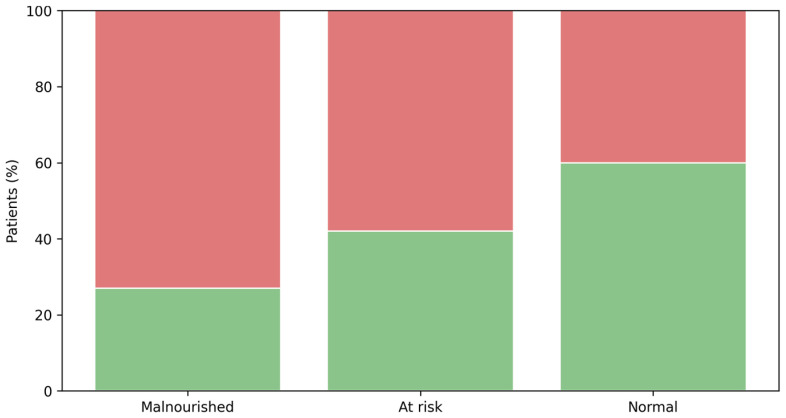
Occult malnutrition according to the MNA category. Prevalence of occult malnutrition across MNA categories (MNA < 17; 17–23.5; ≥24). Green: No; Red: Yes. Occult malnutrition differs significantly across categories (Pearson χ^2^ = 7.642, *p* = 0.022).

The prevalence of occult malnutrition differed significantly across MNA categories (Pearson χ^2^ = 7.642, *p* = 0.022). Occult malnutrition was present in 72.7% (16/22) of patients classified as malnourished (MNA < 17), in 58.3% (56/96) of those at risk of malnutrition (MNA 17–23.5), and in 40.7% (22/54) of patients with a non-pathological MNA score (MNA ≥ 24) (Figure 1). These findings indicate that, even among patients with a normal nutritional screening profile, approximately two in five met criteria for biological vulnerability consistent with the occult malnutrition definition.

### 3.3. Clinical Outcomes According to Occult Malnutrition Status

Serum albumin showed a significant inverse correlation with length of hospital stay (r = −0.201, *p* = 0.008), inversely correlated with C-reactive protein (r = −0.218, *p* = 0.004) and positively correlated with hemoglobin levels (r = 0.249, *p* = 0.001). Length of hospital stay was inversely correlated with hemoglobin (r = −0.234, *p* = 0.002), whereas its correlation with CRP did not reach statistical significance (r = 0.148, *p* = 0.053). No significant correlation was observed between CRP and hemoglobin (r = −0.090, *p* = 0.239).

Occult malnutrition was associated with prolonged hospitalization. In the unadjusted analysis (Table 2, Model 1), prolonged LOS occurred in 56/94 (59.6%) patients with occult malnutrition compared with 30/78 (38.5%) without occult malnutrition, yielding an unadjusted odds ratio (OR) of 2.358 (95% CI 1.275–4.360). In multivariable logistic regression adjusting for age ≥ 85 years, sex, and primary reason for hospital admission, occult malnutrition remained independently associated with prolonged LOS (adjusted OR 2.141, 95% CI 1.136–4.036; *p* = 0.019). Age ≥ 85 years (OR 1.275, 95% CI 0.651–2.496; *p* = 0.479), sex (male vs. female: OR 1.940, 95% CI 0.858–4.388; *p* = 0.111), and admission reason category (OR 0.913, 95% CI 0.773–1.079; *p* = 0.286) were not independently associated with prolonged LOS.

When the MNA score was additionally included in the model (Table 2, Model 2), occult malnutrition remained a significant independent predictor of prolonged LOS (adjusted OR 2.036, 95% CI 1.071–3.870; *p* = 0.030). In this fully adjusted model, MNA was not independently associated with prolonged hospitalization (OR 0.956 per 1-point increase, 95% CI 0.875–1.046; *p* = 0.328), and the associations of age ≥ 85 years, sex, and admission reason category with LOS remained non-significant. These findings indicate that the biological phenotype of occult malnutrition confers prognostic information beyond conventional nutritional screening.

In the interaction model (Table 2, Model 3) occult malnutrition remained independently associated with prolonged length of stay (adjusted OR 1.98, 95% CI 1.01–3.88; *p* = 0.048). No significant interaction was observed between global comorbidity burden and primary reason for hospital admission (adjusted OR for interaction 1.13, 95% CI 0.92–1.37; *p* = 0.245), indicating that the effect of comorbidity on length of stay was consistent across admission categories. Age ≥ 85 years, sex, and admission reason were not independently associated with prolonged hospitalization in this model. ADL was not included in multivariable models because it was assessed only at discharge and could be influenced by length of stay itself, potentially introducing reverse causation.

### 3.4. Subgroup Analysis: Patients with Non-Pathological MNA (MNA ≥ 24)

In the subgroup with nutritionally normal MNA (n = 54), occult malnutrition was present in 22/54 (40.7%). In this subgroup, the association between occult malnutrition and prolonged LOS was not statistically significant in the adjusted model including age ≥ 85 years and sex (OR 1.577, 95% CI 0.484–5.143; *p* = 0.450). Age ≥ 85 years (OR 0.839, 95% CI 0.264–2.668; *p* = 0.766) and sex (male vs. female: OR 2.280, 95% CI 0.647–8.029; *p* = 0.200) were also not significant predictors of prolonged LOS. Multivariable analysis including age ≥ 85 years, sex, and global comorbidity burden showed no statistically significant association between occult malnutrition and prolonged length of stay (adjusted OR 1.31, 95% CI 0.38–4.52; *p* = 0.665). These findings do not exclude a prognostic role but suggest limited statistical power in this subgroup.

The dependent variable was prolonged length of stay, defined as hospitalization beyond the 75th percentile of the cohort. Age ≥ 85 years was modeled dichotomously, sex as male versus female, and primary reason for admission as a categorized variable derived from free-text diagnoses. Global comorbidity burden was assessed using the cumulative CIRS-G score, which was standardized (z-score) prior to inclusion in the model to reduce collinearity. An interaction term between standardized CIRS-G and admission reason was included to test for effect modification. Odds ratios (ORs) and 95% confidence intervals (CIs) are reported.

## 4. Discussion

Collectively, this study shows that occult malnutrition is highly prevalent in very old, hospitalized patients. It identifies a biological vulnerability often missed by standard nutritional screening. Occult malnutrition is associated with prolonged length of stay, independent of age, comorbidities, and MNA. Chronological age alone does not independently predict prolonged hospitalization. Simple biological markers, instead, provide clinically relevant prognostic information.

The present cohort represents a very old, hospitalized population, with a mean age close to 87 years and a substantial burden of clinical complexity. Within this context, occult malnutrition was highly prevalent, affecting more than half of the study population (54.7%), despite the absence of overt clinical malnutrition in a substantial proportion of cases. This finding is clinically meaningful. The prevalence observed in our cohort is higher than typically reported when malnutrition is assessed exclusively through clinical screening tools or anthropometric criteria. It suggests that occult malnutrition may capture a hidden biological vulnerability that may remain undetected by standard nutritional assessments alone. Our results align with emerging evidence indicating that biological derangements often precede clinically apparent malnutrition and may represent an early manifestation of impaired metabolic and inflammatory homeostasis in very old adults [15,16]. Patients with occult malnutrition were slightly older than those without (87.8 vs. 85.5 years), and age as a continuous variable differed significantly between groups. However, age ≥ 85 years was not independently associated with prolonged length of stay in any multivariable model. This observation reinforces a key concept in contemporary geriatric medicine: chronological age alone is an insufficient proxy for biological vulnerability [17,18]. Instead, age-related outcomes appear to be mediated by biological and functional derangements that reflect reduced physiological reserve. In this study, occult malnutrition emerged as a potential determinant of adverse hospital trajectories more than advanced age itself.

Patients with occult malnutrition experienced significantly longer hospitalizations, with a mean difference of approximately four days compared to those without occult malnutrition. Moreover, the prevalence of prolonged LOS was markedly higher in the occult malnutrition group (59.6% vs. 38.5%). Importantly, occult malnutrition remained independently associated with prolonged LOS across all multivariable models. The strength and consistency of this association—adjusted for age, sex, admission reason, MNA, and global comorbidity burden—underscore the importance of the finding. The adjusted odds ratios ranged from 1.98 to 2.14, indicating that patients with occult malnutrition had approximately a twofold higher risk of prolonged hospitalization, independent of traditional clinical and nutritional variables. Moreover, while patients with occult malnutrition tended to have a higher cumulative comorbidity burden (CIRS-G), this difference approached but did not reach statistical significance. Importantly, adjustment for comorbidity burden and inclusion of an interaction term between CIRS-G and admission reason did not materially attenuate the association between occult malnutrition and prolonged LOS. This finding suggests that occult malnutrition is not merely a surrogate of comorbidity severity, but rather an independent biological condition with direct clinical consequences.

A substantial proportion of patients classified as “at risk” or even “nutritionally normal” according to MNA still exhibited occult malnutrition. Crucially, when both occult malnutrition and MNA score were included in the same multivariable model, only occult malnutrition retained an independent association with prolonged LOS, while MNA did not. The predominance of patients classified as “at risk” rather than frankly malnourished by the MNA is consistent with previous reports from acute geriatric settings [19]. Although the MNA is a validated and widely used screening tool, it was originally developed for relatively stable populations and relies heavily on anthropometric, functional, and subjective components that may be transiently preserved or difficult to interpret during acute illness. Several studies have highlighted the limited sensitivity of screening tools in detecting inflammation-driven or disease-related malnutrition in hospitalized older adults [20].

Approximately two-fifths of patients classified as nutritionally normal by MNA exhibited biological vulnerability. However, in this subgroup, the association between occult malnutrition and prolonged length of stay did not reach statistical significance after adjustment. This finding should be interpreted cautiously, as subgroup analyses were exploratory and limited by sample size, resulting in wide confidence intervals. Rather than excluding a prognostic role, these results suggest that the impact of biological vulnerability in patients with preserved screening status may be heterogeneous and context dependent. Further studies are necessary to test such a hypothesis.

The decision to define occult malnutrition using a composite of serum albumin, hemoglobin, and CRP was biologically grounded and supported by the extensive literature linking these markers to adverse outcomes in hospitalized older adults. Hypoalbuminemia is a well-established predictor of morbidity, prolonged hospitalization, and mortality, reflecting the combined effects of inflammation, capillary leak, and catabolic stress rather than nutritional intake alone [21,22,23]. Anemia, highly prevalent in older populations, is consistently associated with reduced physiological reserve, functional decline, and poorer outcomes across medical and surgical settings, independent of classical nutritional deficiency [24,25,26]. CRP was included to capture systemic inflammatory burden, a central driver of metabolic dysregulation, muscle catabolism, and impaired recovery during acute illness. Elevated CRP has repeatedly been associated with prolonged hospitalization and reduced responsiveness to nutritional interventions, consistent with the malnutrition–inflammation complex syndrome [19,27,28]. Thus, together, albumin, hemoglobin, and CRP operationalize a multidimensional construct of biological vulnerability that extends beyond traditional definitions of malnutrition. This approach aligns with contemporary geriatric models emphasizing physiological reserve, resilience, and homeostatic capacity rather than isolated disease states or static nutritional measures [19,20]. By requiring the coexistence of multiple abnormalities, the present definition enhances specificity and identifies patients with a more consolidated biological burden [15,16].

The present study addresses a clinically relevant and underexplored dimension of nutritional vulnerability in acutely hospitalized older adults by proposing a biologically grounded composite phenotype rather than relying on isolated biomarkers or traditional screening tools alone. The use of routinely available laboratory parameters enhances clinical applicability and reproducibility. Integration of biological vulnerability with functional, clinical, and comorbidity measures enabled a comprehensive assessment of patient complexity in a real-world geriatric setting. The consistent association between occult malnutrition and clinically meaningful outcomes, including prolonged length of stay, across multiple adjusted models supports the potential prognostic value of this approach. Some limitations should be acknowledged. First, its observational design and single-center setting limit causal inference and may reduce external validity. The relatively small sample size further constrains statistical power, particularly for subgroup and interaction analyses. Second, no formal dietary assessment was available, and neither sarcopenia, polypharmacotherapy, nor body composition measures were systematically evaluated, precluding a comprehensive characterization of nutritional status beyond biochemical. In addition, inflammatory status was assessed at a single time point, preventing evaluation of longitudinal inflammatory trajectories during hospitalization. Finally, inflammation may interfere with the interpretation of albumin as a nutritional marker; however, in this study albumin was intentionally considered as an indicator of biological vulnerability rather than nutritional intake, in line with its known behavior during acute illness.

## 5. Conclusions

In conclusion, occult malnutrition, as defined, is a common and underrecognized biological vulnerability in very old, hospitalized patients. Identified through simple routine biomarkers, it provides prognostic information beyond age, comorbidity burden, and standard nutritional screening. Integrating biological vulnerability screening into geriatric care pathways may improve early risk stratification and support more individualized interventions to optimize hospital outcomes.

## Figures and Tables

**Table 1:** Baseline characteristics of the study population according to occult malnutrition status.

	All Population (N = 172)	No Occult Malnutrition (N = 78)	Occult Malnutrition (N = 94)	*p*-Value
**Demographic and clinical characteristics**				
Age, years	86.7 ± 6.0	85.45 ± 6.16	87.79 ± 5.61	0.01
Age ≥ 85 years	114/172 (66.3%)	47/78 (60.3%)	67/94 (71.3%)	0.128
Sex, female	139/172 (80.8%)	64/78 (82.1%)	75/94 (79.8%)	0.707
Length of hospital stay, days	12.5 ± 8.9	10.29 ± 6.15	14.31 ± 10.27	0.002
Prolonged length of stay (LOS > 75th percentile)	86/172 (50.0%)	30/78 (38.5%)	56/94 (59.6%)	0.006
**Nutritional assessment (Mini Nutritional Assessment)**				
MNA score	21.2 ± 3.6	21.92 ± 3.43	20.61 ± 3.74	0.017
Malnourished (MNA < 17)	22/172 (12.8%)	6/78 (7.7%)	16/94 (17.0%)	0.022
At risk of malnutrition (MNA 17–23.5)	96/172 (55.8%)	40/78 (51.3%)	56/94 (59.6%)	
Normal nutritional status (MNA ≥ 24)	54/172 (31.4%)	32/78 (41.0%)	22/94 (23.4%)	0.013
**Biological parameters**				
Albumin, g/dL	3.64 ± 0.72	3.93 ± 0.57	3.41 ± 0.75	<0.001
Hypoalbuminemia (<3.5 g/dL)	73/172 (42.4%)	11/78 (14.1%)	62/94 (66.0%)	<0.001
Hemoglobin, g/dL	12.03 ± 1.85	13.13 ± 1.44	11.12 ± 1.65	<0.001
Anemia (Hb < 12 g/dL)	80/172 (46.5%)	17/78 (21.8%)	63/94 (67.0%)	<0.001
C-reactive protein, mg/dL	3.92 ± 5.19	1.73 ± 3.04	5.74 ± 5.88	<0.001
Elevated CRP (>0.5 mg/dL)	124/172 (72.1%)	34/78 (43.6%)	90/94 (95.7%)	<0.001
**Functional and psycho-cognitive status**				
ADL at discharge	2.85 ± 2.14	3.27 ± 2.09	2.49 ± 2.13	0.018
IADL at discharge	2.27 ± 2.77	2.64 ± 2.87	1.96 ± 2.66	0.112
Corrected MMSE	23.1 ± 4.3	23.00 ± 4.28	23.15 ± 4.29	0.895
Geriatric Depression Scale (GDS)	5.12 ± 2.28	5.11 ± 2.38	5.14 ± 2.19	0.939
**Comorbidity burden**				
CIRS-G cumulative score	14.4 ± 5.1	13.59 ± 5.25	15.10 ± 4.96	0.055
CIRS severity index	1.11 ± 0.39	1.05 ± 0.40	1.16 ± 0.38	0.055

Data are presented as mean ± standard deviation or number (%), as appropriate. *p*-values from independent samples *t*-test for continuous variables and χ^2^ test for categorical variables.

**Table 2:** Multivariable logistic regression for prolonged LOS.

**Model 1**	**Adjusted OR**	**95% CI**	***p*** **-value**
Occult malnutrition	2.141	1.136–4.036	0.019
Age ≥ 85 years	1.275	0.651–2.496	0.479
Sex (male vs. female)	1.940	0.858–4.388	0.111
Admission reason category	0.913	0.773–1.079	0.286
**Model 2**	**Adjusted OR**	**95% CI**	***p*** **-value**
Occult malnutrition	2.036	1.071–3.870	0.030
Mini Nutritional Assessment	0.956	0.875–1.046	0.328
Age ≥ 85 years	1.232	0.627–2.424	0.545
Sex (male vs. female)	1.978	0.873–4.482	0.102
Admission reason category	0.907	0.767–1.073	0.254
**Model 3**	**Adjusted OR**	**95% CI**	***p*** **-value**
Occult malnutrition	1.975	1.007–3.876	0.048
Age ≥ 85 years	1.124	0.553–2.281	0.747
Sex (male vs. female)	1.550	0.656–3.662	0.318
Admission reason category	0.907	0.756–1.087	0.291
CIRS-G cumulative score (z-score)	1.326	0.673–2.611	0.415
CIRS-G × admission reason interaction	1.125	0.923–1.372	0.245

## Data Availability

The datasets used/or analyzed during the current study will be available from the corresponding author upon reasonable request.

## Supplementary Materials

The following supporting information can be downloaded at: https://www.mdpi.com/article/10.3390/nu18040586/s1, Table S1: Examples of free-text admission diagnoses and corresponding categorized admission reason.

## References

[1] Kirkland L.L., Kashiwagi D.T., Brantley S., Scheurer D., Varkey P. (2013). Nutrition in the Hospitalized Patient. J. Hosp. Med..

[2] Correia M.I.T.D., Hegazi R.A., Higashiguchi T., Michel J.P., Reddy B.R., Tappenden K.A., Uyar M., Muscaritoli M. (2014). Evidence-Based Recommendations for Addressing Malnutrition in Health Care: An Updated Strategy from the FeedM.E. Global Study Group. J. Am. Med. Dir. Assoc..

[3] Norman K., Pichard C., Lochs H., Pirlich M. (2008). Prognostic Impact of Disease-Related Malnutrition. Clin. Nutr..

[4] Vellas B., Guigoz Y., Garry P.J., Nourhashemi F., Bennahum D., Lauque S., Albarede J.L. (1999). The Mini Nutritional Assessment (MNA) and Its Use in Grading the Nutritional State of Elderly Patients. Nutrition.

[5] Cederholm T., Jensen G.L., Correia M.I.T.D., Gonzalez M.C., Fukushima R., Higashiguchi T., Baptista G., Barazzoni R., Blaauw R., Coats A. (2019). GLIM Criteria for the Diagnosis of Malnutrition—A Consensus Report from the Global Clinical Nutrition Community. Clin. Nutr..

[6] Damanti S., Senini E., De Lorenzo R., Merolla A., Santoro S., Festorazzi C., Messina M., Vitali G., Sciorati C., Rovere-Querini P. (2024). Acute Sarcopenia: Mechanisms and Management. Nutrients.

[7] Santulli G., Sabatelli G., Wang B., Savino M., Bruno F.P., Jankauskas S.S., Massaro A., Peluso C., Vicario M., Savino L. (2025). Interplay Between Frailty and Cardiometabolic Disorders: From Pathophysiology to Clinical Implications. Cardiovasc. Diabetol..

[8] Cabrerizo S., Cuadras D., Gomez-Busto F., Artaza-Artabe I., Marín-Ciancas F., Malafarina V. (2015). Serum Albumin and Health in Older People: Review and Meta Analysis. Maturitas.

[9] Helminen H., Luukkaala T., Saarnio J., Nuotio M. (2017). Comparison of the Mini-Nutritional Assessment Short and Long Form and Serum Albumin as Prognostic Indicators of Hip Fracture Outcomes. Injury.

[10] Li S., Zhang J., Zheng H., Wang X., Liu Z., Sun T. (2019). Prognostic Role of Serum Albumin, Total Lymphocyte Count, and Mini Nutritional Assessment on Outcomes After Geriatric Hip Fracture Surgery: A Meta-Analysis and Systematic Review. J. Arthroplast..

[11] Keller U. (2019). Nutritional Laboratory Markers in Malnutrition. J. Clin. Med..

[12] Coelho-Júnior H.J., Calvani R., Picca A., Tosato M., Russo A., Landi F., Marzetti E. (2025). Biomarkers Linked to Malnutrition Identified According to GLIM Criteria Among Older Community-Dwelling Adults: Results from the IlSIRENTE Study. Nutrients.

[13] Clegg A., Young J., Iliffe S., Rikkert M.O., Rockwood K. (2013). Frailty in Elderly People. Lancet.

[14] Mancinetti F., Guazzarini A.G., Gaspari M., Croce M.F., Serra R., Mecocci P., Boccardi V. (2025). Integrating Nutrition, Inflammation, and Immunity: The CALLY Index as a Novel Prognostic Biomarker in Acute Geriatric Care. Nutrients.

[15] Norman K., Haß U., Pirlich M. (2021). Malnutrition in Older Adults—Recent Advances and Remaining Challenges. Nutrients.

[16] Carriere I., Dupuy A.M., Lacroux A., Cristol J.P., Delcourt C. (2008). Biomarkers of Inflammation and Malnutrition Associated with Early Death in Healthy Elderly People. J. Am. Geriatr. Soc..

[17] Salignon J., Rizzuto D., Calderón-Larrañaga A., Zucchelli A., Fratiglioni L., Riedel C.G., Vetrano D.L. (2023). Beyond Chronological Age: A Multidimensional Approach to Survival Prediction in Older Adults. J. Gerontol. Ser. A Biol. Sci. Med. Sci..

[18] Shakeel H., Jin D., Jin K. (2026). Biological Age Should Anchor Age-Related Disease Research. Aging Dis..

[19] Volkert D., Beck A.M., Cederholm T., Cereda E., Cruz-Jentoft A., Goisser S., de Groot L., Großhauser F., Kiesswetter E., Norman K. (2019). Management of Malnutrition in Older Patients—Current Approaches, Evidence and Open Questions. J. Clin. Med..

[20] Cederholm T., Barazzoni R., Austin P., Ballmer P., Biolo G., Bischoff S.C., Compher C., Correia I., Higashiguchi T., Holst M. (2017). ESPEN Guidelines on Definitions and Terminology of Clinical Nutrition. Clin. Nutr..

[21] Allison S.P., Lobo D.N. (2024). The Clinical Significance of Hypoalbuminaemia. Clin. Nutr..

[22] Vincent J.-L., Dubois M.-J., Navickis R.J., Wilkes M.M. (2003). Hypoalbuminemia in Acute Illness: Is There a Rationale for Intervention?. Ann. Surg..

[23] Soeters P.B., Wolfe R.R., Shenkin A. (2018). Hypoalbuminemia: Pathogenesis and Clinical Significance. JPEN J. Parenter. Enteral Nutr..

[24] Penninx B.W.J.H., Guralnik J.M., Onder G., Ferrucci L., Wallace R.B., Pahor M. (2003). Anemia and Decline in Physical Performance among Older Persons. Am. J. Med..

[25] Peng S., Wu Q., Wang X., Yang J., Wang X., Wei J., Shi W., Zhang X., Wang B. (2025). Joint Association of Low Muscle Mass and Mild Anemia with All-Cause, Cardiovascular and Cancer Mortality Among US Middle and Older Adults. BMC Public Health.

[26] Carmel R. (2001). Anemia and Aging: An Overview of Clinical, Diagnostic and Biological Issues. Blood Rev..

[27] Capurso C., Lo Buglio A., Bellanti F., Serviddio G. (2025). C-Reactive Protein/Albumin Ratio vs. Prognostic Nutritional Index as the Best Predictor of Early Mortality in Hospitalized Older Patients, Regardless of Admitting Diagnosis. Nutrients.

[28] Şahin C., Şahin Y. (2026). Prognostic Significance of the C-Reactive Protein–Albumin–Lymphocyte Index and the Pan-Immune-Inflammation Value in Ischemic and Hemorrhagic Stroke: A Comparative Analysis of Subtypes. Front. Neurol..

