# Supplementary material for: Beyond the MNA: A Biological Vulnerability Phenotype Associated with Prolonged Hospitalization in Older Adults
Source: Nutrients. 2026 Feb 11;18(4):586. doi: 10.3390/nu18040586 (PMC12943111; doi:10.3390/nu18040586)
Supplement: Supplementary file 1 [file nutrients-18-00586-s001.zip › nutrients-4133926-supplementary.pdf]

Table S1. Examples of free-text admission diagnoses and corresponding categorized admission reason

Free-text admission diagnosis (original record)	Key terms identified	Assigned admission category	Rationale
"Fall and trauma"	Fall	Fall/Trauma	Primary driver of hospitalization was fall with or without traumatic injury
"Fever, dyspnea, elevated CRP – suspected pneumonia"	fever, infection, pneumonia	Infectious / Febrile / Sepsis-related	Infectious condition identified as the main acute clinical issue
"Acute heart failure exacerbation with pulmonary congestion"	heart failure, pulmonary	Cardio-respiratory (non-infectious)	Non-infectious cardiovascular decompensation
"Confusion, syncope"	confusion, syncope	Neurological / Cognitive / Syncope-related	Neurological presentation predominated over other comorbidities
"Anemia with fatigue and functional decline"	anemia, fatigue	Metabolic / Renal / Anaemia / Failure-to-thrive	Anemia identified as the main cause of admission and clinical deterioration

Notes: Classification was based on the primary clinical driver of hospitalization, as documented by the admitting physician. Secondary diagnoses and comorbidities were not used for category assignment unless they clearly represented the main reason for admission.

Textual flowchart describing the decision process used to classify primary reason for hospital admission

1. Start from the free-text admission diagnosis recorded in the medical chart.
2. Standardize text (lowercase conversion, removal of non-informative characters).
3. Identify key clinical terms (e.g., infection, fracture, heart failure, anemia, confusion).
4. Is there a single dominant acute condition clearly responsible for hospitalization?
    - Yes → Assign the corresponding admission category.
    - No / multiple conditions present → Proceed to step 5.
5. Apply hierarchical priority rules based on the main clinical driver of acute care needs:
    - Trauma
    - Infection / sepsis requiring antimicrobial or intensive treatment
    - Acute cardio-respiratory decompensation
    - Neurological / cognitive acute presentation (e.g., syncope, delirium)

- Metabolic, renal, anemia-related, or failure-to-thrive conditions

6. Assign the admission category corresponding to the highest-priority condition identified.

Purpose of categorization: This approach was designed to account for diagnostic heterogeneity and approximate the clinical context of acute hospitalization, rather than to quantify disease severity.
